# Carbon Dots as Potent Antimicrobial Agents

**DOI:** 10.7150/thno.39863

**Published:** 2020-01-01

**Authors:** Xiuli Dong, Weixiong Liang, Mohammed J. Meziani, Ya-Ping Sun, Liju Yang

**Affiliations:** 1 Department of Pharmaceutical Sciences, Biomanufacturing Research Institute and Technology Enterprise, North Carolina Central University, Durham, NC 27707, USA; 2 Department of Chemistry and Laboratory for Emerging Materials and Technology, Clemson University, Clemson, South Carolina 29634, USA; 3 Department of Natural Sciences, Northwest Missouri State University, Maryville, Missouri 64468, USA

**Keywords:** carbon dots, antimicrobial, light activation, photodynamic effect, reactive oxygen species, multi-drug resistance.

## Abstract

Carbon dots (CDots) have emerged to represent a highly promising new platform for visible/natural light-activated microbicidal agents. In this article, the syntheses, structures, and properties of CDots are highlighted, representative studies on their activities against bacteria, fungi, and viruses reviewed, and the related mechanistic insights discussed. Also highlighted and discussed are the excellent opportunities for potentially extremely broad applications of this new platform, including theranostics uses.

## Introduction

Infectious diseases caused by microorganisms such as bacteria, fungi, viruses, or parasites, are a leading cause of death worldwide. Many of these diseases become more challenging to treat if the pathogens further develop to become multidrug-resistant (MDR), resulting in increasing mortality rates and higher medical costs. Additionally, the resistance issues are no longer confined to some specific pathogenic species and to only some selected healthcare facilities, but in fact affect virtually all major pathogens and all types of epidemiological settings, including acute-care hospitals, long-term-care facilities, communities, etc., with the observation of considerable variability in the epidemiology of resistance 1, 2. MDR bacterial infections, especially those caused by Gram-positive bacteria have evolved at an alarming rate and developed resistance to nearly all currently available antibacterial treatments. For example, methicillin-resistant *Staphylococcus aureus* (MRSA), vancomycin-resistant *Enterococcus faecium* (VRE), and drug-resistant *Streptococcus pneumoniae* have been identified as serious public threats by United States Centers for Disease Control and Prevention (CDC) 3. The rise in MDR by pathogens has also been accompanied by a continuous decline in the discovery and development of new antibiotics, posing profound challenges globally. There are predictions such that by 2050 the acceleration of antibiotic resistance could cause 300 million additional deaths and cost an extra US$100 trillion, which demands urgent and effective actions to address the MDR issue.

Among the approaches to inactivate pathogenic microorganisms and prevent infections in hospitals and other healthcare facilities, a wide range of disinfectants and antiseptics are commonly used. However, most of these disinfectants are extremely irritating and toxic, resulting in health complications such as contact dermatitis and mucous membrane irritation, and some of them are becoming less effective because of the bacteria's adaptation and resistance 4. Therefore, there is an urgent and growing need for the discovery and development of alternative antimicrobial strategies and antimicrobial agents with superior properties and less toxicity for the prevention and treatment of infections to counter MDR. In this regard, antimicrobial photodynamic inactivation (PDI) has shown great potential in the inactivation of many known classes of microorganism, with major advantages such as minimal invasiveness, low occurrence of side effects, and suitability for rapid and repetitive application 5-11. The PDI treatment is less likely to induce the development of resistance by the targeted microorganisms, as it is based on nonspecific oxidative damages to biomolecules (lipids, proteins, and nucleic acids) in the cellular membrane or within the cells by reactive oxygen species (ROS). The formation of ROS in PDI is through the excitation of photosensitizers by harmless light of appropriate wavelength. The ROS may include radical ions such as superoxide (^•^O_2_^‒^), hydroxyl radical (^•^OH), and lipid derived ions, and/or singlet oxygen (^1^O_2_), and their productions are mechanistically understood as being associated with the type I and/or type II photodynamic effects, respectively 6, 8.

**Figure 1:**
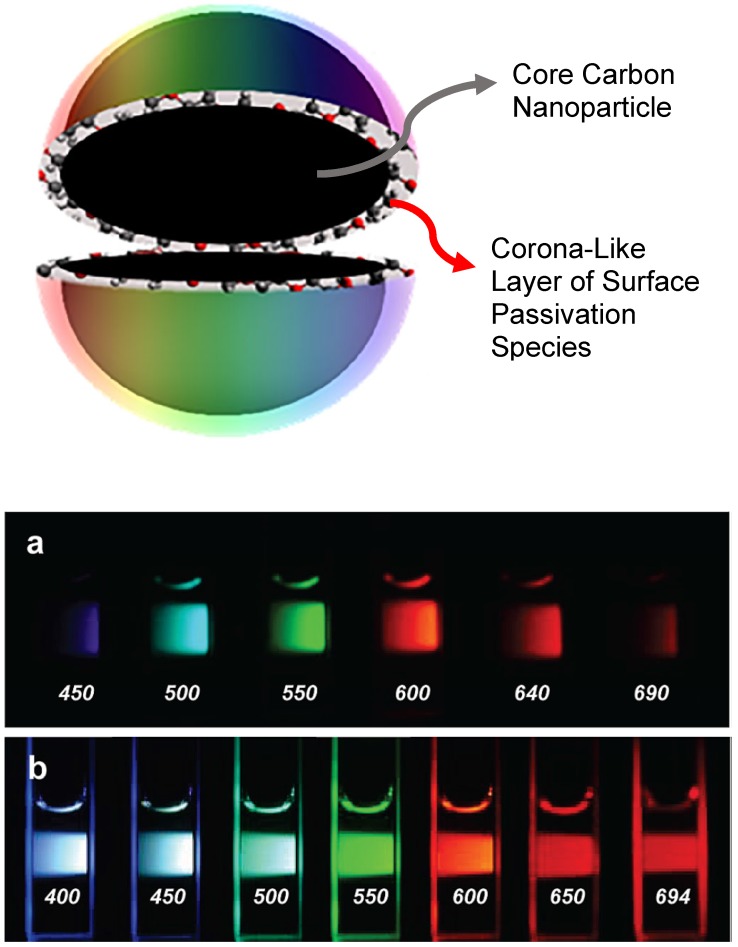
Upper: Cartoon illustration on a carbon dot, which is generally a small carbon nanoparticle core with attached and strongly adsorbed surface passivation molecules (a configuration similar to a soft corona). (Reprinted with permission from ref. 30) Lower: Aqueous solution of a representative sample of CDots (a) excited at 400 nm and photographed through band-pass filters of different wavelengths as indicated, and (b) excited at the indicated wavelengths and photographed directly. (Reprinted with permission from ref. 31).

Among popular photosensitizers have been dye molecules such as porphyrins, phenothiazines, phthalocyanines, bacteriochlorins, and their various derivatives 7, 8. In more recent development, nanoscale materials have emerged as excellent alternative antimicrobial agents by serving not only as vehicles to improve the selective delivery and dispersion of photosensitizers in targeted cells, but also as photosensitizers themselves to enhance the effectiveness of PDI. While nanoscale metal particles and semiconductors have been widely explored for such a purpose, carbon nanomaterials for their broad optical spectral coverage and other advantageous materials characteristics have also attracted much recent attention in PDI related applications 12-15. Beyond the excitements associated with the famous nanoscale carbon allotropes (fullerenes, nanotubes, and graphenes), the recent recognition of carbon nanoparticles as a distinct zero-dimensional carbon allotrope, *versus* fullerenes as “zero-dimensional all carbon molecules” of defined chemical stoichiometry and structures, has created new opportunities in the continuing fight against pathogens and MDR. By exploiting and enhancing the intrinsic optical properties and photoinduced redox characteristics of carbon nanoparticles, carbon dots (CDots) 16-26, which are generally defined as small carbon nanoparticles of various surface passivation schemes (Figure 1) 17-19, have emerged to represent a new platform for visible/natural light-activated antimicrobial agents 12-15. In this regard, CDots are essentially unique visible photosensitizers for effective PDI, with additional benefits due to their advantageous properties including the nontoxic nature 18, 19, 27-29, photostability, versatility in surface functionality for desired microbial adhesion and interactions, and their production from abundant and inexpensive precursors for extremely broad and low to ultralow cost applications.

In this article, we highlight several aspects of CDots as relevant to their visible/natural light-activated microbicidal properties, review some of the representative studies of CDots' antimicrobial activities, and provide perspectives on the challenges and opportunities in both fundamental development and practical uses of this new class of antimicrobial agents.

## 2. Carbon Dots (CDots)

**Figure 2:**
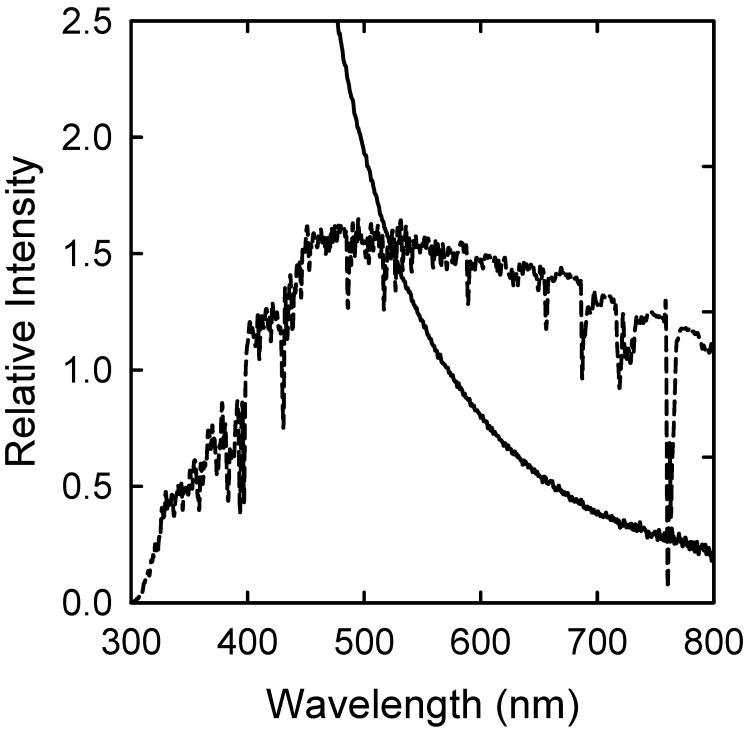
The observed absorption spectrum of broadly distributed CDots in aqueous solution (solid line), which is essentially the same as that of the aqueous dispersed carbon nanoparticles used for the CDots, except for less scattering effects, and the comparison with the solar spectrum at the sea level (dashed line). (Reprinted with permission from ref. 39)

CDots (Figure 1) 20, 21, 23, 32, 33, also referred to in some literature as carbon quantum dots despite the lack of any convincing evidence for the classical quantum confinement effect in these nanomaterials, were discovered originally for their bright and colorful fluorescence emissions that resemble those found in conventional semiconductor quantum dots (QDs). For photoexcitation, the optical absorptions of CDots are dictated by electronic transitions in the core carbon nanoparticles of the dots, which are known to be associated with π-plasmons in the nanoparticles. The carbon nanoparticles may be largely amorphous or more graphitic, including those often referred to as “graphene quantum dots” in the literature 17, 20, 34-38. The absorptions are relatively strong, covering a broad spectral region of near-UV and visible, extending into the near-IR (Figure 2). More quantitatively in terms of optical absorptivities, the absorptions of CDots can be quantified by absorptivity values per molar concentration of carbon atoms (M_C-atom_) in the core carbon nanoparticles. As determined experimentally for the carbon nanoparticles of 4-10 nm in diameter, the absorptivity values are 50-100 M_C-atom_^-1^cm^-1^ at 400-450 nm, compared with 16 M_C-atom_^-1^cm^-1^ for C_60_ at its first absorption band maximum 24. Therefore, CDots can readily be activated by visible light or even under ambient room lighting conditions.

**Figure 3:**
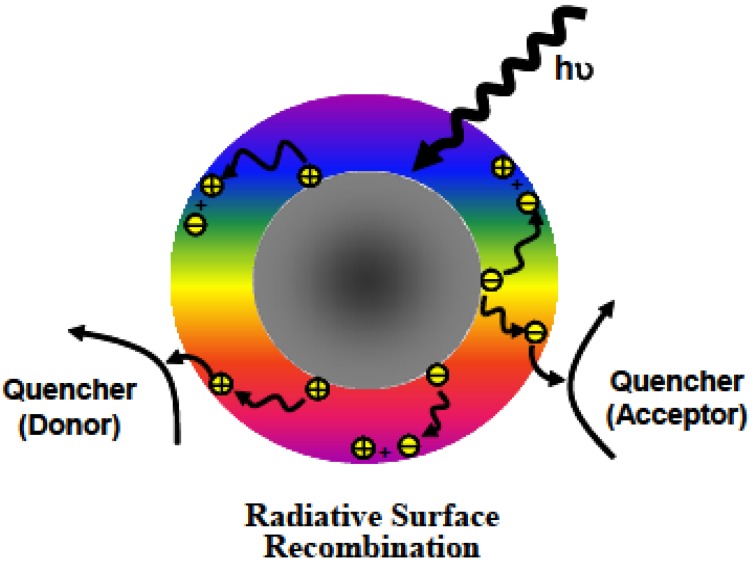
Cartoon illustration on the mechanistic framework for photoexcited state processes in CDots, such that upon photoexcitation of the core carbon nanoparticles in the dots there is rapid charge separation to form electrons and holes, which are localized at the passivated surface defect sites, and the radiative recombinations of the electrons and holes are responsible for the observed fluorescence emissions. The ROS generation might be associated with the charge separated species and/or the emissive excited states. (Reprinted with permission from ref. 39)

According to available experimental results, the photoexcited CDots undergo rapid charge separation for the formation of electrons and holes, which are trapped at various surface sites stabilized by the surface passivation. The observed bright and colorful fluorescence emissions are attributed to radiative recombinations of the electrons and holes, phenomenologically similar to those in conventional semiconductor QDs. The photo-generated electrons and holes have been explored successfully for uses in reductive and oxidative reactions, respectively (Figure 3) 16, 17, 22, 24-26, 39-41. The photoexcited state processes and redox characteristics of CDots must also be responsible for their observed photodynamic effects 16, 17, 22, 24-26, 39-45 and antimicrobial properties 12-15.

**Figure 4:**
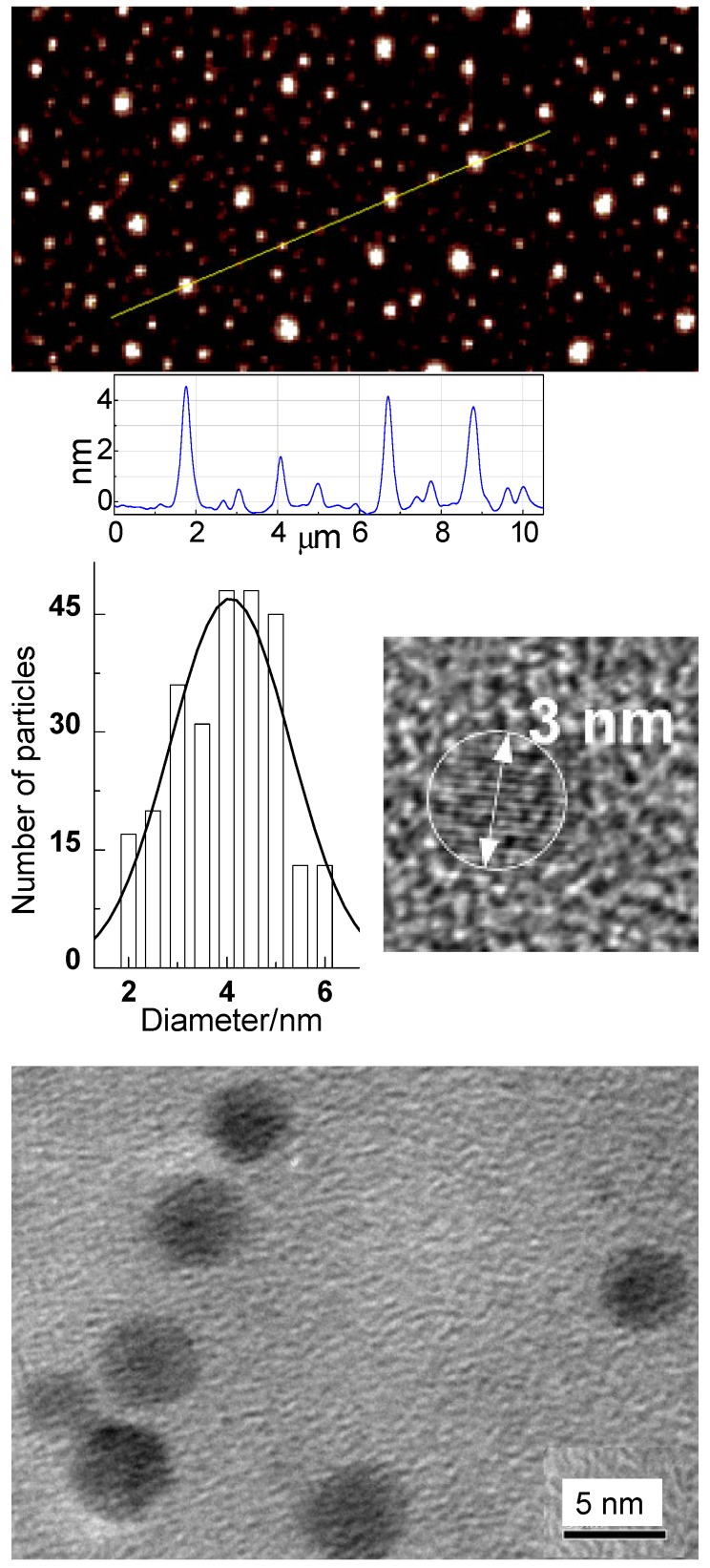
Top: Atomic force microscopy (AFM) imaging results for EDA-CDots on mica substrate, with height profiles of some dots along the line highlighted; Middle: (left) The size distribution based on height analyses of multiple AFM images, fitted with the Gaussian distribution curve, and (right) a high-resolution transmission electron microscopy (TEM) image illustrating the carbon core in a dot; Bottom: TEM images of the EDA-CDots. (Reprinted with permission from ref. 19)

The originally reported CDots were prepared by chemically functionalizing the surface of pre-processed and selected small carbon nanoparticles with selected organic and polymeric molecules 20, 21, and the synthesis has since been classified as the deliberate chemical functionalization method. For the use of pre-existing carbon nanoparticles, the dot structures from such a synthesis are obviously more closely aligned with the general definition of CDots. Shown in Figure 4 are some characterization results for the well-established EDA-CDots, in which the small carbon nanoparticles are surface passivated by the functionalization with 2,2-(ethylenedioxy)bis(ethylamine) (EDA) molecules, suggesting that the structures and chemical compositions of CDots are relatively simple.

**Figure 5:**
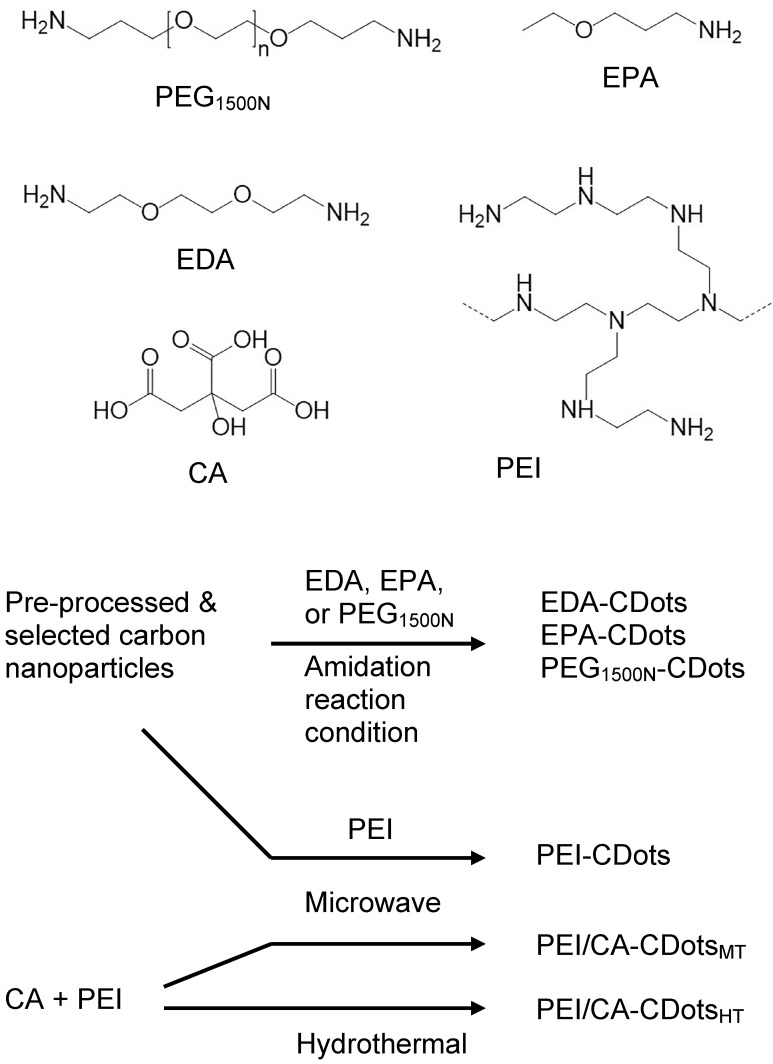
Selected organic molecules used for surface functionalization and/or as precursors for the various CDots, and the different reaction schemes for syntheses of the CDots under different processing conditions, including the chemical functionalization (amidation) and thermally induced functionalization of pre-existing carbon nanoparticles, and the carbonization via microwave or hydrothermal processing. (Reprinted with permission from ref. 30)

In more recent studies, the pre-processed and selected small carbon nanoparticles were also surface-functionalized by organic species in thermal reactions, with the functionalization achieved through the melting of a portion of each organic species onto the carbon nanoparticle surface for the desired passivation effect 40, 46. Such a synthesis based on pre-existing carbon nanoparticles also produces CDots that are structurally more closely aligned with the general definition (Figure 1). Shown in Figure 5 are some representative CDots prepared by the synthetic methods described above, whose antimicrobial properties are highlighted in next sections.

A more popular method for the preparation of CDots has been the thermal carbonization of organic or other carbon-rich/containing precursors, often in "one-pot" processing, with or without the involvement of a solvent 18, 19, 37, 41, 47-50. An implicit assumption with the carbonization synthesis in relationship to the general definition on CDots is such that the carbonization of organic precursors for the formation of core carbon nanoparticles is incomplete, with the remaining organic species attached to or associated with the formed nanoscale carbon domains serving the required surface passivation function. The carbonization synthesis is obviously convenient and versatile, which may be credited for their great popularity, accounting for overwhelming majority of the CDots reported in the literature. Recently, however, there have been serious concerns on the structures and compositions in some of the dot samples prepared by one-pot carbonization 37, 41, 47-52, especially those processed under rather mild experimental conditions such as “cooking some mixtures of organic species” at a temperature up to 200 °C for only a few hours 30, 32, 47. Insufficient carbonization and significant contamination of dye-like species generated in the thermal processing may prove problematic for those samples in their various intended applications, including antimicrobial uses. Extreme caution is advised in the selection of processing conditions for the carbonization synthesis.

## 3. Killing/Inhibition of Bacteria

**Figure 6:**
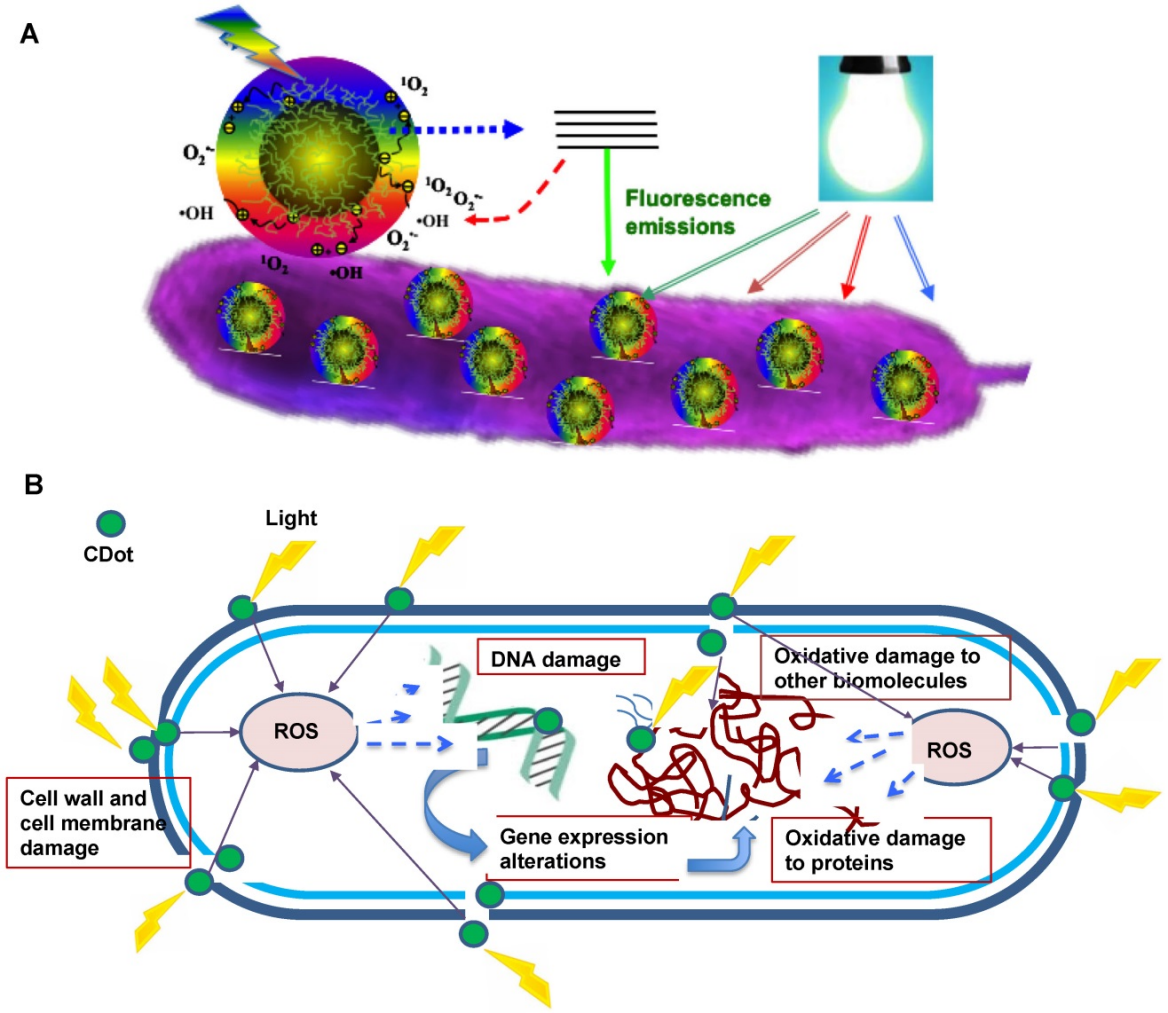
** Cartoon illustration on the mechanism of action for CDots' photoactivated antibacterial activities. (A)** Adhesion of CDots to bacterial surface, and visible light-induced generation of ROS. (Reprinted with permission from ref. 62) **(B)** Intracellular ROS cause damages to bacterial cell.

CDots are generally known as benign and nontoxic *in vitro* and *in vivo*. However, with their effective light-harvesting over a very broad spectral range from UV to near-IR, CDots have exhibited strong photodynamic effects, with relevant uses in cancer therapy reported 53-57. Similarly, photoexcited CDots are capable of producing reactive oxygen species (ROS), which are known to kill/inhibit microorganisms. According to existing research results, the major processes responsible for the antimicrobial effects of CDots are likely associated with the generation of ROS. As depicted in Figure 6, the mechanism of action includes the adhesion of CDots to the bacterial surface, the photoinduced production of ROS, the disruption and penetration of the bacterial cell wall/membrane, the induction of oxidative stress with damages to DNA/RNA, leading to the alterations or inhibitions of important gene expressions, and the induction of oxidative damages to proteins and other intracellular biomolecules 12, 58, 59. Under visible/natural light illumination, CDots in contact with the bacteria cell can efficiently generate ROS by activating the oxygen in air or water, leading to the production of hydroxyl free radicals (OH^•^) and/or singlet oxygen (^1^O_2_), which can destroy some of the critical biomolecules in cell and lead to cell death 60, 61. It is well-documented that ROS induces intracellular protein inactivation, lipid peroxidation, dysfunction of the mitochondria, and gradual disintegration of the cell membrane, followed by necrosis/apoptosis and eventual cell death. While mechanistic details on the generation of ROS by different types of CDots and their corresponding antibacterial activities are yet to be understood, the available experimental results have shown great promise for CDots as a new class of effective antibacterial agents with ready activation by visible/natural light.

**Figure 7:**
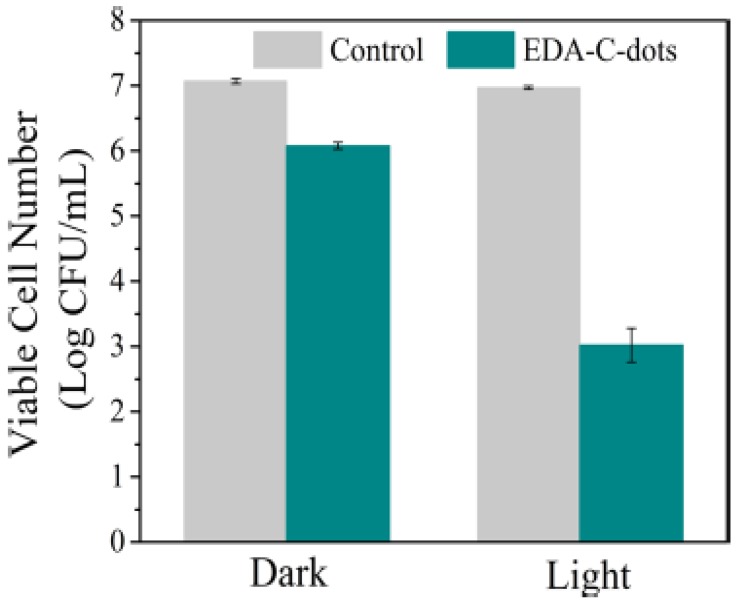
Reductions in the viable cell number after E. coli cells were treated with EDA-CDots for 30 min with or without light. (Reprinted with permission from ref. 15)

In the first report on the visible/natural light-activated antibacterial activities of CDots 15, the multi-institution collaborative team of researchers demonstrated that EDA-CDots (Figure 4) under visible/natural light could effectively inhibit *E. coli* cells both in suspensions and on the agar surface. Experimentally, when *E. coli* cells were treated under white light for 30 min, the viable cell number reduced ~4 logs, while the same treatment in the dark as control only reduced ~1 log (Figure 7). In the same study, significant inhibition of *E. coli* growth in medium and reduction in colony number on agar plates due to the EDA-CDots treatment with light activation were also observed. These antibacterial activities were attributed mechanistically to photodynamic effects, similar to those reported earlier in the killing of cancer cells by CDots under photoirradiation 55.

Several subsequent studies of CDots from different syntheses yielded results that were consistent with those described above. For example, Li, *et al*. synthesized CDots by a one-step electrochemical method with vitamin C as precursor, and found their broad-spectrum antibacterial activities against *S. aureus*, *B. subtilis*, *Bacillus sp*. WL-6, *E. coli*, and the ampicillin-resistant *E. coli* 58. For example, the CDots at a concentration of 50 µg/mL could inhibit the growth of *B. subtilis* and *Bacillus sp*. WL-6 cells. Hou, *et al*. used the antibiotic ciprofloxacin hydrochloride as precursor to prepare CDots in hydrothermal processing at 200 °C, with the stated purpose for the mild processing conditions to retain some ciprofloxacin-like structure on the dot surface 63. The MIC value of the dot sample was lower for *E. coli* than that for *S. aureus*, suggesting poorer antibacterial effect of the sample on Gram-positive bacteria than on Gram-negative bacteria. Penicillin was also used as precursor for CDots in similar hydrothermal carbonization processing, but at a surprisingly low temperature of only 120 °C 64. For comparison, CDots were also prepared from similar processing of a different precursor mixture containing no penicillin, followed by the attachment of penicillin to the dot surface 64. The two versions of CDots, which should both contain penicillin but probably in different structural arrangements, were evaluated for their antibacterial activity against *Staphylococcus aureus, E. coli* (DH5α), MDR *E. coli*, and methicillin-resistant *Staphylococcus aureus* (MRSA). The positive results obtained under visible light were attributed to the maintenance of penicillin and/or the ROS generation 64.

There have been serious recent questions on how to define the materials obtained from the carbonization processing under conditions unlikely to produce a sufficient amount of carbon in the nanoscale carbon domains that are required for CDots. Higher processing temperatures coupled with longer treatment times have produced samples of properties more comparable with those of CDots from the chemical functionalization of pre-existing carbon nanoparticles 30, 47. With the use of hydrothermal carbonization at 250 °C for 8 h, Liu, *et al*. used metronidazole as precursor for CDots 65. There was seemingly a purpose to have the metronidazole structure completely changed in the resulting dots with the more aggressive processing conditions, which was confirmed in the study according to the authors, thought at the same time it might also defeat the purpose of carbonizing an antibiotic instead of some inexpensive organic species. Nevertheless, the as-prepared CDots from metronidazole could not inhibit the growth of *S. mutans* or *E. coli*, but displayed selective antibacterial activity against only obligate anaerobes 65.

As generally expected, the antibacterial activities of CDots are dependent on many parameters, including the optical properties, photoexcited state characteristics, and surface functionalities of CDots, and the dependencies provide opportunities to manipulate and enhance their visible/natural light-activated bactericidal functions.

***Light Activation***. PDI is a photoinduced process, so the light source must be compatible with the optical absorptions of CDots in terms of maximal wavelength overlap. CDots are broadly and strongly absorptive over the visible spectrum, extending into the near-IR (Figure 2). The absorptions are due primarily to transitions associated with π-plasmons in the core carbon nanoparticles of the dots, as discussed in the previous section. The broad and strong optical absorptions of CDots are responsible for their antimicrobial properties under visible light, including household light/natural ambient light. For example, Awak, *et al*. used EDA-CDots (Figure 4) to treat *B. subtilis* cells, and found that the treatment in the dark and under room light for 1 h resulted in a similar magnitude of viable cell reduction, at approximately 1 log (~90%), while under the laboratory light (visible light from a 36 W LED lamp at a distance of 10 cm away from the top surface of the plate), the same treatment resulted in 2 log (~99%) viable cell reduction. When the treatment time was increased to 3 h, the bactericidal effects in the dark and under room light did not increase significantly, but the bacterial killing by the same treatment under the laboratory light increased dramatically to approximately 4 logs (~99.99) 12.

In other reported studies, Kováčová, *et al*. synthesized hydrophobic CDots and embedded them in polyurethane and polydimethylsiloxane matrices to examine the polymeric composite surface for antibacterial activities, and found singlet oxygen generation on the surface upon irradiation by blue light, corresponding to up to 5 logs of inhibition effects against *S. aureus*, *E. coli*, and *K. Pneumoniae* with up to 60 min of the light irradiation 66. Similarly in other antimicrobial studies with CDots, blue and visible light were used. A limitation with these light wavelengths is their suitable only for treating superficial layers of infected tissues due to their generally poor penetrability and significant absorption by haemoglobin and water molecules in tissues. For deep light penetration into tissues for potential applications of PDI *in vivo*, longer wavelength light sources are necessary, which require the development of CDots with high absorption coefficients in the biological transparency window over the red to near-IR spectral region (650-950 nm). Host-guest CDots with selected red/near-IR dyes encapsulated/embedded in the core carbon nanoparticles of CDots have been suggested as a promising solution 67, 68.

CDots are also known for their record-setting multiphoton absorption cross-sections in the near-IR, on the order of 40,000 GM (Goeppert-Mayer unit, 1 GM = 1 × 10^-50^ cm^4^ s/photon) at 800 nm 69, which offers an alternative in the quest to extend the light activation into the longer wavelength region for the bactericidal properties of CDots. In a recent study 70, the two-photon excitation in the near-IR (800 nm) was exploited for the generation of ROS and the associated antibacterial activities against Gram-positive and Gram-negative bacteria.

***Photoexcited State Properties***. Again, PDI is photoinduced, so the light-activated bactericidal activities of CDots are correlated with or dictated by the photoexcited state processes and redox characteristics of the dots. CDots are also known for their bright and colorful fluorescence emissions, which may be employed as a convenient and effective tool in the understanding of the correlation between the photoexcited state properties and the antibacterial outcomes. Among the various fluorescence parameters, quantum yields are readily measured experimentally to provide relatively quantitative insight into the competition of fluorescence with other excited state processes responsible for photodynamic effects.

**Figure 8:**
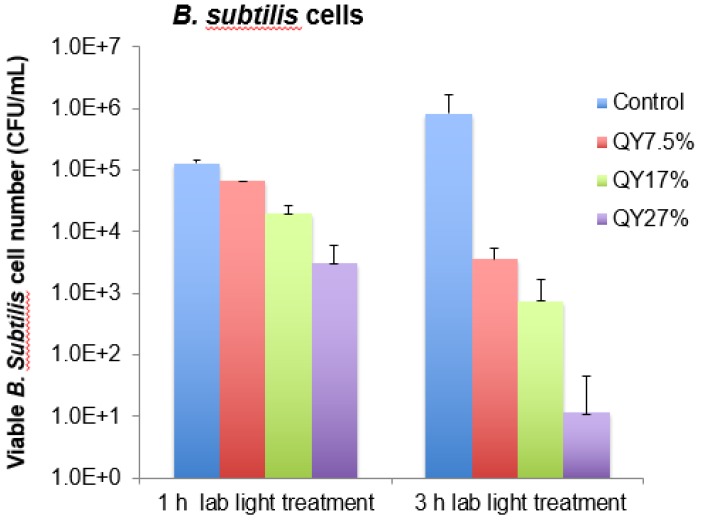
The viable cell reduction of *B. subtilis* cells after the treatments with EDA-CDots samples with fluorescence quantum yields of 7.5%, 17%, and 27% at 15.8 μg mL^-1^ under lab light for 1 h and 3 h. (Reprinted with permission from ref. 12).

Al Awak,* et al*. examined the visible light-activated antibacterial function of a series of EDA-CDots with fluorescence quantum yields (Φ_F_) ranging from 7.5% to 27%, which were obtained by the gel column fractionation of the as-synthesized EDA-CDots sample 12. The results revealed a correlation of antimicrobial activities of CDots with their observed Φ_F_ values, showing a clear trend of decreasing viable cell numbers for *B. subtilis* cells upon treatments with EDA-CDots of increasing Φ_F_ values under otherwise the same conditions (Figure 8). More quantitatively, upon the 3 h treatment with EDA-CDots of 7.5%, 17%, and 27% in Φ_F_ under visible light illumination, the reductions in viable *B. subtilis* cells were about 2.4, 3.1, and 4.9 logs, respectively 12. In the same study, it was also found that the magnitudes in viable cell reduction for *E. coli* cells were significantly lower than those for *B. subtilis* cells under the same treatment conditions, suggesting that the antibacterial function of CDots varied with bacterial species. Similar variations have been reported in the literature on the use of more traditional antimicrobial agents against Gram-negative and Gram-positive bacteria 12.

***Effects of Dot Surface Functionalities***. Surface functionalization of nanomaterials is generally an important strategy in affecting the interactions between the nanomaterials and biological entities, determining the pathway of cellular uptake, and influencing intracellular trafficking and cytotoxicity of the nanomaterials 71. CDots are structurally versatile, with much flexibility in surface modifications to impart properties tailored for specific applications, such as changing the surface charge status/characteristics and optical properties in particular. The surface functional groups and charge of CDots could have critical effects on their interactions with bacterial cells, influencing their antibacterial actions substantially.

In a recent study Abu Rabe, *et al*. looked into the role of surface functionalization of CDots in their antibacterial function, and found that the surface charge played a critical role in interactions of the dots with bacterial surfaces, which are essential to the desired effective antibacterial activities 62. EDA-CDots and EPA-CDots (Figures 4 & 5) were evaluated to determine and compare the effect of different terminal groups/charges on the light-activated antibacterial activities. EDA and EPA (Figure 5) are small molecules of similar chemical structures, except for their bearing two and one amine group (-NH_2_), respectively, and consequently their corresponding CDots differ in terminal groups/charges on the dot surface. EDA-CDots with the surface primary amine moieties tend to be positively changed at physiological pH (the formation of -NH_3_^+^), whereas EPA-CDots with surface methyl (-CH_3_) terminal groups are not charged. Both CDots had similar average diameter (4-5 nm) and fluorescent quantum yields (~20%), but different antibacterial outcomes. The 1 h treatment with 0.1 mg/mL EDA-CDots with light illumination for *B. subtilis* cells showed much greater antibacterial activity compared to the treatment with EPA-CDots under the same conditions. The former resulted in about 3.26 log reduction in viable cells, while the latter barely showed any reduction. The results were rationalized by the presence of strong binding-like interactions between the positively charged terminal groups (-NH_3_^+^) on EDA-CDots and the negatively charged bacterial surface, thus locally a high concentration of EDA-CDots on bacterial surface for the observed more effective antibacterial actions 62. The same surface functional moiety and charge effects were also found in the antibacterial activities of CDots functionalized by oligomeric polyethylenimine (PEI-CDots, Figure 5).

Bing, *et al*. synthesized CDots of three different surface functionalities, including spermine for positively charged, candle soot-derived for negatively charged, and glucose for neutral, and investigated the surface effect on the inhibition of *E. coli* 72. They found no effect on bacterial growth with neutral dots, but bacteria apoptosis with both the negatively and positively charged dots 72. The reported results suggested different levels of ROS generation by the three dots of different surface charges, the positively charged being most effective and then the negatively charged. However, the reported inability for the neutral dots to generate ROS is surprising, suggesting that there might be effects with the three samples other than the dot surface charge-based interactions with the targeted bacterial cells, because there is no evidence for such interactions to be a requirement for the ROS generation by CDots.

The effects of dot surface functionalities do go beyond the charge status and associated interactions with bacterial cells. The thickness of the corona-like surface passivation layer on individual CDots (Figure 1) may also affect their antibacterial efficiency. For example, CDots functionalized with oligomeric polyethylenimine (PEI) of different sizes, 1,200 vs. 600 in average molecular weight, were evaluated for their light-activated antibacterial activities 62. These two types of CDots were structurally rather similar, except for their difference in the thickness of the surface passivation layer due to the size of the PEI used for the surface functionalization of pre-existing carbon nanoparticles. The treatment with 0.1 mg/mL PEI_600_-CDots under visible light illumination for 1 h inactivated almost all the cells in the samples, with >7 log viable cell number reduction for *B. subtilis* cells, while the treatment with PEI_1200_-CDots under the same conditions only resulted in 1.82 log reduction in viable cell number. The much higher antibacterial efficiency of PEI_600_-CDots could be rationalized such that the corresponding thinner passivation layer might allow the ROS to act more effectively on the bacterial cells for the observed more effective antibacterial function 62.

***Hybrid CDots and Other Modifications***. There has been much effort in the search for ways to enhance the light-activated antibacterial function of CDots. One popular approach has been the modification of CDots on the surface with molecules that would enhance the affinity to or interactions with bacterial cells, conceptually similar to the manipulation of dot surface charge discussed above. For example, Li, *et al*. modified CDots with spermidine to introduce positive surface charge and to explore the known characteristics of spermidine in binding with negatively charged DNA, lipids, and proteins 59. The modified CDots exhibited antibacterial activities against *E. coli*,* S. aureus*, *B. subtilis*, and *P. aeruginosa* and also some MDR bacteria, such as methicillin-resistant *S. aureus* (MRSA). The inhibition effect was attributed to the multivalent interaction of the dots with the negatively charged bacterial membrane through binding to multiple nucleic acids, proteins, porins, and/or peptidoglycans. Surprisingly, however, the ROS assay suggested no intracellular ROS generation.

Carbon-based hybrid nanostructures, especially carbon hybrid dots with metal and metal oxides in the dot structure, have been explored to enhance the antibacterial activities found in the parent CDots. For hybrid CDots, nanoscale carbon has been successfully coupled with various nanoscale semiconductors, such as TiO_2_ 73, ZnO 74, Na_2_W_4_O_13_/WO_3_ 75, Cu_2_O 76, and others 77, 78. Some of these carbon-based/derived hybrid dots showed significantly enhanced activities against pathogenic bacteria under UV/vis photo-irradiation, which were attributed to more efficient charge transfers and the suppressing effect on the recombination of electron-hole pairs for enhanced ROS generation. In particular, neat TiO_2_ nanoparticles are known for their photocatalytic properties, and they have been used for antibacterial and general disinfection purposes to take advantage of their chemical stability, high specific surface area, low toxicity, and the ability to produce charge carriers under UV irradiation 79-81. However, colloidal TiO_2_ is also known for its required UV excitation due to the inherent large bandgap energy and the relatively fast recombination of electron-hole pairs. The former represents a major limitation for the more desirable application conditions of visible/natural light activation, and the latter corresponds to less effective ROS generation and weaker antibacterial performance. Since the nanoscale carbon in CDots harvests visible photons effectively, the carbon/TiO_2_ hybrid dots should be able to extend the light activation into the visible spectrum to drive the photocatalytic processes of both nanoscale carbon and TiO_2_ in the dot structure. Liu, *et al*. found that in carbon/TiO_2_ hybrid dots the structural configuration for the arrangement between the nanoscale carbon and TiO_2_ domains in the core of individual dots played a critical role in the observed optical and photoinduced redox properties 82. Overall, however, the properties of the carbon/TiO_2_ hybrid dots were similar to those found in dye-sensitized TiO_2_ systems, namely for the carbon domains to serve as the dye function of photon-harvesting in the visible spectral region where TiO_2_ nanoparticles have no absorptions, and for the harvested photon energies to subsequently sensitize the TiO_2_ in the hybrid dots 82. Yan, *et al*. prepared CDots decorated with TiO2 via hydrothermal synthesis, and showed that their antibacterial activities against *E. coli* and *S. aureus* compared favourably to those of neat TiO_2_, with the results rationalized as being due to the improved dispersibility, high absorption of visible light, and increased content of ROS under visible light irradiation for the nanoscale carbon - TiO_2_ combination 73.

**Figure 9:**
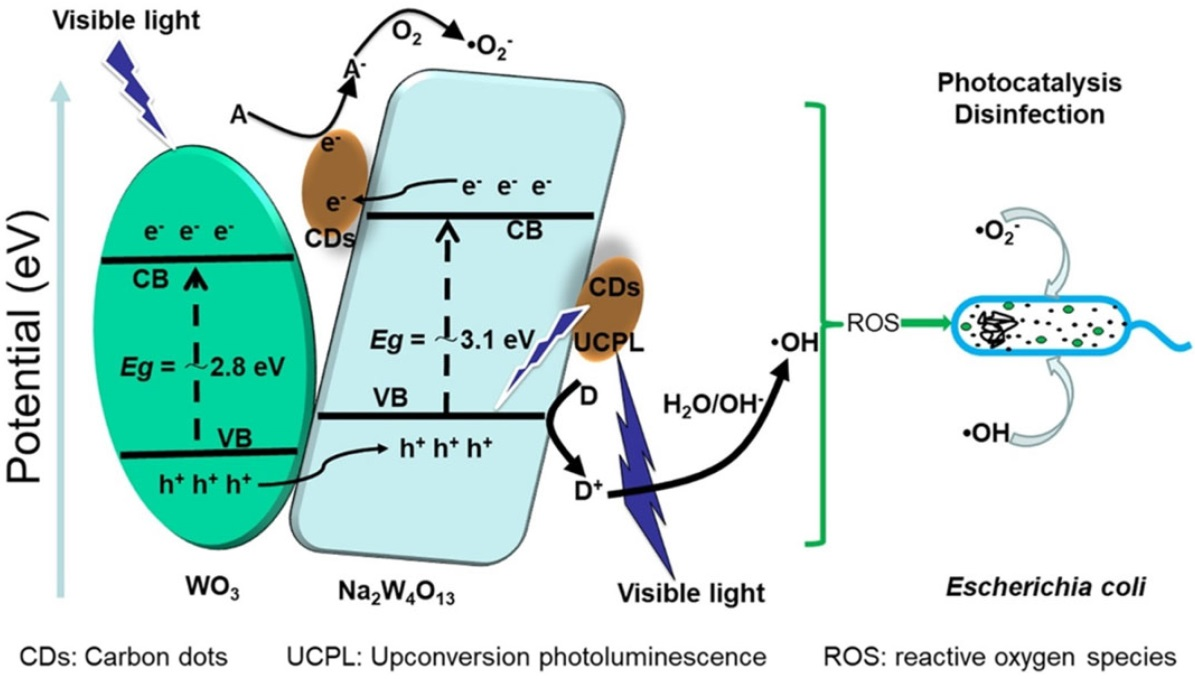
Conceptual illustration on the composition of carbon dots with tungsten oxides for photocatalytic disinfection applications. (Reprinted with permission from ref. 75)

Zhang, *et al*. decorated CDots with Na_2_W_4_O_13_/WO_3_ flakes for their serving as environmentally friendly photo-disinfection material (Figure 9), and found that it was possible to use the material for 7 log reduction of *E. coli* cells under visible light irradiation in 100 min, compared to the 1 log and 2 log reductions by WO_3_/Na_2_W_4_O_13_ and WO_3_ 75. The enhanced photocatalytic disinfection performance was attributed to the photocatalytic production of some ROS species, which were detected in electron spin resonance (ESR) spectroscopy and reactive species scavenging experiments.

CDots have also been incorporated into hydrogel and other matrices for the development of antibacterial surfaces. In addition to the work by Li, *et al*. 58 highlighted above, Xiang, *et al*. decorated an injectable folic acid-conjugated polydopamine hydrogel with carbon-ZnO hybrid nanoparticles, and used the resulting material for wound dressing to accelerate wound healing and control antibacterial activity 83. There were ROS generation and heat production in the hydrogel with 660 nm and 808 nm light illumination, showing activities against *S. aureus* and *E. coli*. Potential use of the material in the reconstruction of bacteria-infected tissues, especially exposed wounds, was proposed 83.

**Figure 10:**
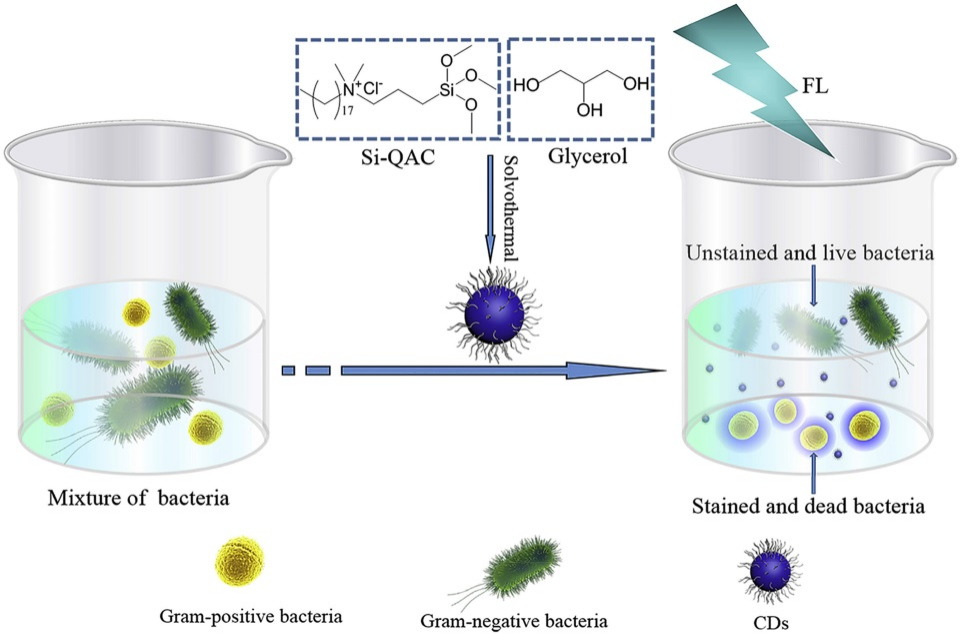
The carbon dots (CDs) with quaternary ammonium moieties could kill Gram-positive bacteria and also stain the dead bacterial cells for fluorescence analyses. (Reprinted with permission from ref. 85).

The CDots platform was also used as essentially a carrier for conventional disinfection agents to kill bacteria and at the same time as a fluorescence label for the analysis of the dead bacterial cells (Figure 10) 84, 85.

***Combination with Other Antimicrobial Reagents***. Over several decades, oxidizing antimicrobial chemicals such as hydrogen peroxide (H_2_O_2_) and sodium hypochlorite (NaOCl) have been used extensively as universal disinfectants against a wide range of microbes 86. However, some bacterial species have shown resistance to the antiseptic action of oxidizing chemicals and thus require the agents to be used in high dosages, which could cause extensive damage to other biological systems such as human tissues. As a solution to the problem, a combination of selected antimicrobial agents with nontoxic photo-activated CDots could be an effective strategy to achieve the desired maximal antibacterial activity at the minimal dosage of individual agents, thus to reduce effectively the potential toxicity posed by antimicrobial chemicals on environments and public health, and to mitigate the development of microbial resistances 86. For such a purpose, Dong, *et al*. recently combined EDA-CDots (Figure 4) with several antimicrobial reagents, including H_2_O_2_, Na_2_CO_3_, and acetic acid, and investigated their ability to achieve synergistic or enhanced antimicrobial effect against bacterial cells 86. The results indicated that the treatment with a combination of CDots and H_2_O_2_ did have synergistic effects in inhibiting the growth of both Gram positive *B. subtilis* and Gram negative *E. coli* cells. More quantitatively on the reduction in viable cell numbers, the treatment with the combination of 10 µg/mL CDots and 8.82 mM H_2_O_2_ resulted in 2.46 log reduction for *E. coli* cells, significantly more than the sum of viable cell reductions achieved by treatments with the individual components of CDots for 0.14 log and H_2_O_2_ for 1.57 log. Such synergistic effect was most likely acting in the way that the antibacterial mechanisms of CDots and H_2_O_2_ enhanced each other by catalyzing the decomposition of H_2_O_2_ to produce more hydroxyl radicals. However, the combination of CDots with Na_2_CO_3_ or acetic acid yielded no synergistic effects, but the negative outcomes actually provided justification from the opposite side on the value of the combination strategy. The strategy is a valid one, just requiring a careful selection of appropriate antimicrobial agents to be combined with CDots to achieve the desired synergistic effects for much enhanced antibacterial performance at minimal dosage of the agents.

Similar to the combination strategy has been the coupling of CDots with conventional antibiotics, in which the dots also serve the role as drug carriers. For example, Jijie, *et al*. attached ampicillin (AMP) to CDots bearing surface amino moieties, and evaluated the dot-AMP “conjugates” as visible light-triggered antibacterial agents 87. The conjugate configuration improved the stability of AMP from that of free AMP, yet still preserved the antibacterial properties of AMP and CDots, as demonstrated by the observed inactivation of *E. coli* cells with visible light illumination 87. There have also been uses of CDots-derived conjugates with antibiotics for purposes such as controlled drug release to avoid the increasing microbial resistance caused by over-dosage of antibiotics 88, 89. For example, CDots were conjugated with ciprofloxacin to regulate the release of ciprofloxacin at a sustained rate, and the conjugates were found to be more biocompatible than free ciprofloxacin, with enhanced antimicrobial activities against both model Gram positive and Gram negative bacteria 90.

## 4. Anti-Fungi Effects

There have been a few reported studies on the anti-fungi activities of CDots. For example, Li, *et al*. found that the CDots prepared to inactivate bacteria also exhibited broad-spectrum antifungal activities against *R. solani* and *P. grisea* 58. Jhonsi, *et al*. also found antifungal effects of CDots against *C. albicans* 61. Similarly, CDots conjugated with ciprofloxacin were tested on *Saccharomyces cerevisiae* yeast cells, with the observed bright green fluorescence emissions attributed to the CDots inside the cells 90. Priyadarshini, *et al*. explored the potential of CDots and their derived conjugates for anti-fungi activities against the fungal pathogen *C. albicans* 78. In another reported study, Bagheri, *et al*. used the treatment with CDots to inhibit the growth of yeast cells (*Pichia pastoris* X33 wild type), which was accompanied by significantly altered yeast cell morphology 91. Interestingly, Rispail, *et al*. combined nanoscale carbon with ZnS, and found that while the resulting nanostructures could be taken up significantly by hypha of fungus *Fusarium oxysporum*, they only had low toxicity to the fungus.

## 5. Antiviral Effects

Studies have been scarce on the use of CDots to inactivate viruses and/or reduce infection rates. Du, *et al*. reported that procine kidney (PK-15) cells and monkey kidney (MARC-145) cells treated with CDots could significantly inhibit the multiplication of pseudorabies virus (PRV) and porcine reproductive and respiratory syndrome virus (PRRSV), respectively, which were used as models for DNA virus and RNA virus, respectively 92. The inhibitory effect of CDots was rationalized as being due to the activation of IFN-α and the production of ISGs, which in turn inhibited viruses' replications 92. Type I interferons (IFNs), including IFN-α and IFN-β, are the best known antiviral innate immune molecules with a powerful antiviral response to viral infection in the human body 93.

**Figure 11:**
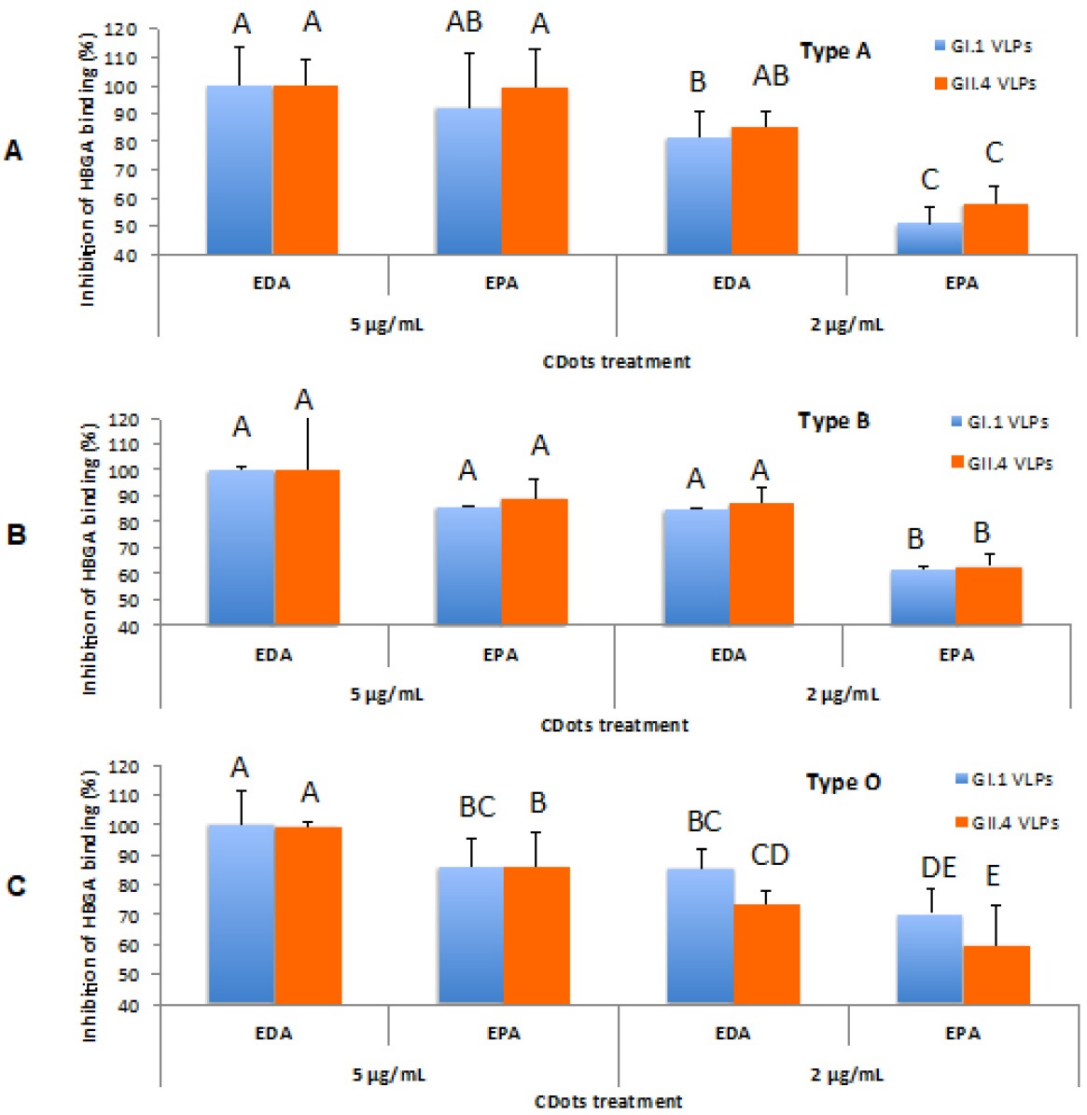
** The inhibitory effects of CDots on NoV VLPs' binding to type A, B, O saliva HBGA receptors. (A)** Type A HBGA; **(B)** Type B HBGA; **(C)** Type O HBGA. Different letters on the columns indicate significant differences at P<0.05, while any same letters on the columns indicate no significant difference. (Reprinted with permission from ref. 94

Dong, *et al*. found that the direct treatment with EDA-CDots or EPA-CDots (Figure 5) for the human noroviruses virus-like-particles (VLPs), GI.1 and GII.4 VLPs, at the dot concentration of 5 µg/mL were highly effective in inhibiting VLPs' binding to histo-blood group antigens (HBGA) receptors on human cells (Figure 11), and moderately inhibiting the binding to their respective antibodies, while without damaging the viral capsid protein and the viral particle morphology 94. The same surface charge effect was also observed as EDA-CDots exhibited more effective inhibitory effect on VLPs' binding to HBGA and their respective antibodies than EPA-CDots did. More recently, Huang, *et al*. found that CDots synthesized from benzoxazine monomer could block the infection of life-threatening flaviviruses (Japanese encephalitis, Zika, and dengue viruses) and non-enveloped viruses (porcine parvovirus and adenovirus-associated virus) *in vitro*, probably via directly binding to the surface of the virion and eventually impeding the first step of virus-cell interaction 95.

Shown in Table 1 is a brief summary of CDots samples and their various antimicrobial uses. These uses may be categorized roughly as follows: (1) CDots and their derived hybrids or composites with visible/natural light activation to kill/inhibit microbials; (2) CDots carrying traditional antibiotics for the light-activated function in (1) to couple with or enhance the effect of the antibiotics; (3) CDots as delivery vehicles for traditional antibiotics or conventional disinfection agents; and (4) others, such as damaging microbial membranes in different mechanisms, inhibition effects due to dot sizes and surface functionalities, and selectively killing Gram-positive bacteria coupled with fluorescence labelling of the dead cells for analyses.

## 6. Theranostics

The increasing findings and improved understanding of the microbicidal properties of CDots have paved the way toward the design of a multifunctional CDots-based theranostic platform for simultaneously integrating the diagnostic functions and therapeutic effects. A few studies have already been reported on using CDots for antimicrobial theranostics to achieve the concurrent effects of bioimaging, drug delivery and release, and photodynamic therapy 90, 96-98. For example, Mitra, *et al*. coated CDots onto the surface of ZnO nanorod for antibacterial and bioimaging applications 97. The resulting nanohybrid could be taken up by *S. aureus* cell to display bright green fluorescence emissions, also exhibiting antibacterial activities against *S. aureus* and *E. coli* in a concentration dependent fashion 97. Thakur, *et al*. reported that CDots could be used simultaneously as a tracer to exploit the photoluminescence properties and as a vehicle to carry and deliver a significant amount of therapeutic agents such as ciprofloxacin for potential theranostics 90. Dou, *et al*. explored CDots for both antibacterial activities and gene delivery capabilities, and observed much enhanced gene transfection efficiency over that of the naked DNA delivery 96. Pramanik, *et al*. febricated multi-component nanostructures by combining fluorescent CDots, magnetic nanoparticles, and pardaxin antimicrobial peptides, which were designed for the diagnosis, selective separation, and early disinfection of pathogens on the same material platform 98. In another investigation 99, CDots-like nanostructures were used as two-photon contrast probes for the tracking and localization purposes in the two-photon imaging in a three-dimensional biological environment and concurrently for the generation of ROS to eliminate MDR bacterial species.

## 7. Conclusions and Perspective

The development of resistance by microorganisms to antibiotics and other disinfection agents in general has been and will continue to be a critical challenge to the human living environment, food safety, and healthcare system. In the quest for novel alternative antimicrobial approaches that are not only effective in mitigating the threat of resistant microorganisms but also benign and nontoxic 18, 27, 28, 100-102, CDots have emerged to represent a promising new platform for visible/natural light-activated microbicidal agents. The excellent potential of the CDots platform in the killing/inhibition of bacteria, fungi, and viruses, including some multi-drug resistant species, has been demonstrated in many reported studies, so has been the path towards theranostics uses, as highlighted in this article. Extensive continuing and new efforts on the further development and exploration of the CDots platform and on the mechanistic understanding of the antimicrobial function, leading to rapid and broad advances in the fundamental research and also technological applications, may be envisaged.

In the further development and exploration, abundant caution should be exercised in the preparation of dot samples to be sure that CDots of the defined structures and chemical compositions are indeed produced in the applied synthetic protocols. CDots exploit the intrinsic optical properties and redox characteristics of carbon nanoparticles, so that the dominating content of nanoscale carbon domains in the dot structure must be emphasized and verified before an as-synthesized sample is declared as CDots and used for subsequent antimicrobial and/or other studies. In this regard, the use of pre-existing carbon nanoparticles for CDots obviously represents a safer approach. However, the carbonization of carbon-rich/containing organic precursors has been and will likely continue to be a more popular method for advantages such as convenience and versatility. When using such a method, one must be mindful that the carbonization of organic species generally requires significantly more harsh conditions than “cooking at 160 ºC for an hour or a few hours”, because those rather mild processing conditions are far from being sufficient to convert organic species into nanoscale carbon structures in any substantial fashion, let alone the “crystalline or graphitic nanoparticles” suggested in some reports. In fact, if this were possible, one would be making CDots in pan-frying or barbecuing vegetables, or even better enjoying the taste of CDots when sipping a cup of tea because the tea in that cup is often processed under conditions much more aggressive than those for the one-pot carbonization referred to above. One should also be mindful that carbon nanoparticles can readily be generated from organic matters in an electron microscopy machine equipped with a powerful electron beam of up to 300 kV. The caution emphasized here is critical to the advancement of the CDots research field, in order to avoid the pollution by results obtained with samples that are complex mixtures of dyes, including red-absorptive and/or emissive dyes, with perhaps only a sprinkle of small carbon pieces.

Photons are extremely powerful (450 nm equivalent to ~32,000K in thermal energy) and everywhere. Carbon is abundant and dirt cheap and CDots are intrinsically benign and nontoxic, so that the new platform of visible/natural light-activated antimicrobial agents may ultimately find extremely broad applications in the fight with infectious microorganisms, drug resistant or otherwise.

## Figures and Tables

**Table 1:** Summary of Carbon Dots Samples and Their Antimicrobial Effect/Outcome

CDots and/or Sample Configuration	Light Activation	Microorganisms	Highlight of Antimicrobial Effect/Outcome	Refs
EDA-CDots (from chemical functionalization of CNPs)	Visible light	*E. coli*, *B. subtilis*	EDA-CDots treatment for 30 min reduced ~4 logs of *E. coli* viable cell numbers. The dots (Φ_F_ ~27%) at 16 µg/mL for 3 h reduced 4.7 logs of *B. subtilis* viable cell numbers.	12,15
EDA-CDots, EPA-CDots, PEI_600_-CDots, & PEI_1200_-CDots (all from functionalization of CNPs)	Visible light	*B. subtilis*	EDA-CDots treatment at 0.1 mg/mL for 1 h reduced 3.26 logs of viable cells, while EPA-CDots treatment barely showed any reduction. PEI_600_-CDots and PEI_1200_-CDots treatment at 0.1 mg/mL for 1 h reduced >7 logs and 1.82 logs viable cells, respectively.	62
Dot sample from carbon nanopowders combined with H_2_O_2_	White light	*E. coli*	A mixture of 10 µg/mL dots and 8.82 mM H_2_O _2_ reduced 2.46 logs of viable cells.	86
Dot sample from electrochemical processing of carbon rod and then coupled with TiO_2_	Visible light	*E. coli*, *S. aureus*	The treatment at 1 µg/mL for 24 h inactivated 90.9% *E. coli* and 92.8% *S. aureus*.	73
Dot sample from carbonization synthesis coupled with ZnO in hydrogel	660 nm & 808 nm light	*S. aureus*,* E. coli*	With the dual-light irradiation, inactivated 99.9% of the bacteria.	83
Dot sample with Na_2_W_4_O_13_/WO_3_	Visible light	*E. coli*	The treatment for 100 min inactivated about 2x10^7^ CFU/mL of *E. coli* cells.	75
Dot sample from carbonization in polymer films	Blue light	*S. aureus*, *E. coli*, *K. pneumoniae*	Light irradiation for 60 min caused up to 5 logs of inhibition effects.	66
Dot sample carrying penicillin	Visible light	*S. aureus*, *E. coli* (DH5α), MDR *E. coli*, MRSA	The treatment at 100 µg/mL inhibited more than 50% of MDR *E. coli* and MRSA.	64
Dot sample from carbonization synthesis coupled with ampicillin	Visible light	*E. coli*	The MIC value decreased to 14 µg/mL from free ampicillin of 25 µg/mL.	87
Dot sample carrying ciprofloxacin hydrochloride		*E. coli*, *S. aureus*	The MIC value lower for *E. coli* than that for *S. aureus*.	63
Dot sample carrying metronidazole		*P. gingivalis*	Only selectively inhibiting obligate anaerobes.	65
Dot sample from carbonization of ammonium citric coupled with spermidine		*E. coli*, *S. aureus*, *B. subtilis*, *P. aeruginosa*, MRSA	Antibacterial activities against all of the tested bacteria.	59
Dot sample from carbonization synthesis carrying quaternary ammonium moieties		*S. aureus*	Killing the Gram-positive bacteria and also staining the dead cells for fluorescence analyses.	84,85
				
Dot sample made from vitamin C		*R. solani* & *P. grisea* fungi	The treatment at 300 µg/mL significantly inhabited the growth of the fungi.	58
Dot sample from carbonization synthesis doped with Au		*C. albicans* fungus	Antifungal activity, with MIC_80_ ~250 µg/mL.	78
				
Dot sample from PEG-diamine & ascorbic acid as precursor		Pseudorabies virus, porcine reproductive and respiratory syndrome virus.	Significantly inhibited the multiplication of the viruses.	92
EDA-CDots & EPA-CDots (both from chemical functionalization of CNPs)		Human noroviruses virus-like particles (VLPs)	EDA-CDots and EPA-CDots at 5 µg/mL inhibited 100% and 85-99%, respectively, of the binding of VLPs to histo-blood group antigens receptors on human cells.	94
Dot sample made from benzoxazine monomer		Japanese encephalitis, Zika, and dengue viruses, and porcine parvorius and adenovirus-associated viruses	The dots could directly bind to the surface of the virion, and eventually impede the first step of virus-cell interaction.	95

Abbreviations: CNPs: carbon nanoparticles; EDA: 2,2-(ethylenedioxy)bis(ethylamine); EPA: 3-ethoxypropylamine; PEI: polyethylenimine; MRSA: methicillin-resistant *S. aureus*.
